# B-cell and antibody responses to SARS-CoV-2: infection, vaccination, and hybrid immunity

**DOI:** 10.1038/s41423-023-01095-w

**Published:** 2023-11-10

**Authors:** Dennis Lapuente, Thomas H. Winkler, Matthias Tenbusch

**Affiliations:** 1 Institut für klinische und molekulare Virologie, Universitätsklinikum Erlangen und Friedrich-Alexander-Universität (FAU) Erlangen-Nürnberg, Schlossgarten 4, 91054 Erlangen, Germany; 2 Department of Biology, Division of Genetics, Nikolaus-Fiebiger-Center for Molecular Medicine, Friedrich-Alexander-Universität Erlangen-Nürnberg (FAU), Erlangen, Germany; 3 Medical Immunology Campus Erlangen, Friedrich-Alexander-Universität (FAU) Erlangen-Nürnberg, Schlossplatz 1, 91054 Erlangen, Germany

**Keywords:** SARS-CoV-2, neutralizing antibodies, memory responses, vaccines, IgG4, Viral infection, Antibodies

## Abstract

The emergence of severe acute respiratory syndrome coronavirus 2 (SARS-CoV-2) in 2019 prompted scientific, medical, and biotech communities to investigate infection- and vaccine-induced immune responses in the context of this pathogen. B-cell and antibody responses are at the center of these investigations, as neutralizing antibodies (nAbs) are an important correlate of protection (COP) from infection and the primary target of SARS-CoV-2 vaccine modalities. In addition to absolute levels, nAb longevity, neutralization breadth, immunoglobulin isotype and subtype composition, and presence at mucosal sites have become important topics for scientists and health policy makers. The recent pandemic was and still is a unique setting in which to study de novo and memory B-cell (MBC) and antibody responses in the dynamic interplay of infection- and vaccine-induced immunity. It also provided an opportunity to explore new vaccine platforms, such as mRNA or adenoviral vector vaccines, in unprecedented cohort sizes. Combined with the technological advances of recent years, this situation has provided detailed mechanistic insights into the development of B-cell and antibody responses but also revealed some unexpected findings. In this review, we summarize the key findings of the last 2.5 years regarding infection- and vaccine-induced B-cell immunity, which we believe are of significant value not only in the context of SARS-CoV-2 but also for future vaccination approaches in endemic and pandemic settings.

## Introduction

In late 2019, a cluster of pneumonia cases of unknown etiology was reported from Wuhan, China [1, 2]. It soon became clear that a novel coronavirus was the causative agent, rapidly leading to an increasing number of infections and deaths. The virus is thought to have originated from zoonotic spillover from bats via an intermediate host associated with Huanan Seafood Wholesale Market [3, 4]. In ensuing months, SARS-CoV-2 encountered an immunologically naïve human population, resulting in the most severe pandemic outbreak since the 1918 Spanish Flu.

To date, more than 767 million confirmed cases of coronavirus disease 2019 (COVID-19) and 6.9 million associated deaths have been reported worldwide [5]. As large parts of the human population have developed immunity to the virus through infection and/or vaccination, the pandemic phase is waning. However, SARS-CoV-2 may become a recurrent, seasonal pathogen, requiring induction of durable immunity or periodic booster vaccinations to protect those at risk. Moreover, after SARS-CoV-1 in 2002 and the Middle East respiratory syndrome (MERS) coronavirus in 2012, SARS-CoV-2 is the third zoonotic betacoronavirus infecting the human population in the last two decades, underscoring the fact that we may face newly emerging CoVs in the future. An in-depth understanding of vaccine- and infection-induced immune responses against SARS-CoV-2 is therefore highly relevant for postpandemic mitigation as well as pandemic preparedness.

The majority of symptomatic SARS-CoV-2 infections result in mild to moderate disease with prototypical symptoms of a respiratory infection, including fever, fatigue, and dry cough [6]. However, a significant proportion of infections progress to more severe and critical disease involving dyspnea, acute respiratory distress syndrome (ARDS) or multiorgan failure. The fatality rate has been estimated to be 0.23% but varies considerably across locations, probably reflecting different population characteristics [7]. Older age and comorbidities are important factors contributing to disease severity, but a range of other variables, including sex, race, and socioeconomic status, have also been discussed [8–11]. In addition to acute illness, SARS-CoV-2 infection can lead to persistent health problems affecting multiple organs, which is referred to as long COVID. This inconsistent, multifaceted disease manifestation is estimated to occur in at least 10% of symptomatic infections, but vaccination prior to infection significantly reduces the risk [12–14].

SARS-CoV-2 is a betacoronavirus that encodes four structural proteins from its large positive-sense RNA genome: spike (S), nucleocapsid (N), matrix (M), and envelope (E). Cell entry is mediated by the trimeric S glycoprotein, which binds to its entry receptor angiotensin-converting enzyme 2 (ACE2) [15]. To allow for fusion of the viral and host cell membranes, the S protein must be primed by furin-mediated cleavage at the S1/S2 site, generating S1 and S2 fragments. A second cleavage at the S2’ site by transmembrane protease serine subtype 2 (TMPRSS2) or cathepsin B/L after attachment or endocytosis, respectively, is required to liberate the fusion peptide [15]. Each S protein monomer contains a receptor-binding domain (RBD) that can be buried within the N-terminal domain (NTD; RBD “down”) or exposed (RBD “up”), with only the RBD in the up position being able to interact with ACE2 [15, 16]. Similar to other viral glycoproteins, S has an energetically unfavorable and unstable prefusion conformation that can spontaneously refold into a postfusion state [16, 17]. The postfusion conformation lacks or hides important epitopes for neutralizing antibodies [17]. Insertion of two proline residues into the C-terminal S2 fusion domain (S-2P) stabilizes the S protein in its prefusion conformation to stably expose the RBD [16]. This strategy is widely used in licensed SARS-CoV-2 vaccines.

The S protein is the exclusive target of SARS-CoV-2-specific nAbs and therefore the most relevant antigen for vaccines. Ninety percent of neutralizing activity in convalescent sera is mediated by RBD-specific nAbs [18–23]. However, nAbs targeting other epitopes in the S protein, such as the S1 NTD or regions important for protease cleavage or conformational changes, have been described [24–28]. S2-specific antibodies are mostly nonneutralizing, with exceptions such as those targeting the S2 stem helix or the fusion peptide [26, 29–32]. Nevertheless, the RBD remains the most prominent target for nAbs, and the resulting immune pressure is reflected by an increasing number of escape mutations in the RBD, which is particularly the case for the omicron variant [33, 34].

In general, vaccines were instrumental in controlling the recent pandemic. In Europe and the US, mRNA-, adenoviral vector-, and protein-based vaccines were licensed and demonstrated efficacies between 74% and 95% against symptomatic disease in clinical trials [35–38]. However, protection against infection decreases over time due to waning immunity and the emergence of variants escaping antibody responses. In particular, nAbs induced by the initially licensed vaccines are less potent against newly emerging VOCs, which eventually led to adaptation of the mRNA vaccines [39, 40]. There is clear evidence of vaccine-mediated protection, at least against severe COVID-19, when ancestral or adapted vaccine boosters are administered at appropriate intervals [41, 42]. These and other insights generated in the course of the recent pandemic will help to optimize vaccine strategies to provide long-term immunity against SARS-CoV-2 but may also support pandemic preparedness against emerging CoVs or other viruses.

## Humoral response to infections

Adaptive immunity consists of antigen-specific antibodies and T cells, which fulfill distinct and complementary roles in viral infections. Although antibody responses can prevent infection by neutralizing the viral inoculum, cytotoxic T cells are critically involved in eliminating virus-infected cells. A key feature of the humoral immune response is generation of highly specific and affine immunoglobulins (Ig) to almost any foreign antigen encountered. B cells, particularly B-cell receptors (BCRs), are the source of this staggering adaptability. V(D)J recombination and clonal selection followed by an iterative affinity maturation process results in a broad and flexible B-cell receptor repertoire, eventually giving rise to plasma cells (PCs) that produce the corresponding antibody response [43].

In the bone marrow, precursor B cells undergo somatic recombination by joining individual V, D, and J gene segments that are present in multiple copies [44]. This process generates diversity at the complementary determining regions of the heavy and light chains, creating germline-encoded BCRs. Following positive and negative selection mechanisms that ensure proper functionality and limited self-reactivity, mature B cells migrate to the spleen to form the naïve B-cell pool [45].

B-cell responses in the context of infection or vaccination can be divided into canonical germinal center (GC) and noncanonical extrafollicular (EF) responses. In GC responses, specific B-cell clones expand within the dark zone of the GC. During this expansion, activation-induced cytidine deaminase (AID) activity leads to mutagenesis of the variable region of the BCR, a mechanism termed somatic hypermutation (SHM). De novo-generated B-cell clones then enter the GC light zone and undergo a Darwinian selection process. The most affine BCRs outcompete other clonotypes in capturing antigens from follicular dendritic cells (FDCs) and receive help from CD4^+^ T follicular helper cells (TFHs) via the CD40 ligand and IL-21 to survive and expand [43, 46–48]. The progressive increase in the affinity of BCRs for a given antigen through iterative mutation and selection in dark and light zones is termed affinity maturation. AID not only promotes mutagenesis of the variable region but also induces class-switch recombination toward IgG subclasses (IgG1, IgG2, IgG3, IgG4), IgE, and IgA [49, 50]. To produce these class-switched, affinity-matured immunoglobulins in serum or mucosal fluids, GC B cells must leave the GC as antibody-secreting, short-lived plasmablasts (PBs) or long-lived PCs [51]. However, GC B cells can also differentiate into MBCs that circulate until antigen re-exposure leads to their re-entry into the GC, followed by new rounds of affinity maturation [52, 53]. Compared to PBs or PCs, MBCs appear to leave GCs earlier with a lower level of SHM, possibly creating greater flexibility in the B-cell memory pool for recognition of related or even unrelated antigens [54–56].

In noncanonical EF responses, B cells expand massively upon contact with antigen-presenting FDCs outside of GCs but without involvement of follicular CD4^+^ T cells. These EF B cells rapidly differentiate into short-lived PBs that cause a prompt but short-lived antibody response. Despite a common misconception that EF B cells do not undergo class-switch recombination or SHM, there is considerable evidence for this, as discussed elsewhere [57]. Nevertheless, the extent of SHM and BCR diversification is not comparable to GC responses, resulting in near germline-encoded BCRs and shared, public antibody responses [58].

The result of these qualitatively different B-cell responses is a division of labor. (i) Extrafollicular B-cell responses generate an early antibody response within a few days without time-consuming SHM and without inducing immunological memory in terms of long-term antibody production or MBCs. (ii) GC B cells differentiate into long-lived PCs that produce mature, high-affinity nAbs to provide a first (and often sufficient) layer of immune memory against reinfection. (iii) MBCs produce a new generation of affinity-matured nAbs in the event of breakthrough infections. The affinity of such GC-mediated antibody responses increases with time after a single antigen exposure but also progressively with each new cognate antigen contact. Many infections and vaccines induce persistent, sometimes lifelong, immunity (though this immunity may not be provided exclusively by B-cell or antibody responses). Typical examples are protective immune responses against measles, mumps, and smallpox; in contrast, immunity to respiratory viruses is much less durable and protective [59]. Immunization with inactivated influenza vaccines induces bone marrow PCs that decline significantly within one year of vaccination [60], whereas infection with respiratory syncytial virus does not induce any protective B-cell memory [61]. Thus, humoral immune responses can effectively protect against infection, but respiratory pathogens may have evolved strategies to undermine these mechanisms.

## Antibody responses to primary SARS-CoV-2 infection

Early in the pandemic, the repertoire of commercially available and “in-house” serological methods began to grow, including enzyme-linked immunosorbent assays, chemiluminescent assays, flow cytometry-based assays, point-of-care lateral flow tests, and pseudotype/live virus neutralization assays [15, 62–65]. These methods have become important tools for diagnosing late/postinfection stages, tracing COVID-19 contacts, assessing epidemiologic aspects and evaluating immunity after infection or vaccination.

### Systemic antibody responses

Regardless of disease severity, almost all SARS-CoV-2-infected individuals experience seroconversion within two to four weeks postinfection, with a median of 12 days postsymptom onset (PSO). In some asymptomatic infected individuals, seroconversion might be absent or transient, but cellular immune responses appear to be comparable to those of symptomatic patients [66, 67]. In the case of seroconversion, IgM, IgA, and IgG appear rather contemporaneously [19, 68–72]. This is in contrast to some other viral infections, in which IgM precedes IgG responses by several days [73], but is congruent with findings for SARS-CoV-1 infections [74]. The viral S and N proteins are major targets of this serological response [19, 72, 75]. Serum IgG, IgA, and IgM levels reach a plateau between two and four weeks PSO [68, 71, 72, 76], before IgM and IgA decline to preinfection levels in the majority of patients within three months [68, 77–80]. Iyer et al. reported median times to seroreversion for RBD-specific IgA and IgM of 71 days and 49 days, respectively [68], but there are also reports indicating a low-level persistence of anti-RBD and anti-S IgA for four [81] and eight months [75]. Early reports also suggested rapid decay among IgG responses, with a short half-life of only 36 to 49 days [19, 82, 83], raising concerns about the durability of infection-induced humoral responses. However, this underestimation of IgG persistence may be due to biphasic decay kinetics. Several studies with a longer observation period described an initial rapid decline within the first four months, followed by a more gradual decline in ensuing months [68, 80, 84]. This late and stable plateau phase has been observed for more than eight [80] and eleven [84] months, and most convalescent patients do not experience seroreversion within 10 months postinfection [79]. nAb titers plateau at three to six weeks postinfection and correlate significantly with RBD-specific IgG levels [68, 70, 71, 76].

Stratification of patient cohorts has revealed a strong positive correlation between disease severity and the height of antibody peak levels as well as response duration [75, 76, 79, 83, 85, 86]. Patients with more severe disease have higher levels of S2-, S1-, and RBD-binding antibodies and concomitantly the strongest serum nAb titers at two to four weeks after infection [85]. Hospitalized COVID-19 patients showed higher RBD-specific IgG levels than nonhospitalized convalescents, even after more than 120 days of PSO [75]. This correlation may be the result of higher viral loads and prolonged viral replication in more severely ill patients, resulting in stronger immune responses, though clear evidence for this hypothesis is lacking [87]. In addition, very high serum IgG and IgA titers occur more frequently in critically ill patients and those with SARS-CoV-2-mediated ARDS [88], possibly reflecting EF B-cell and PB responses (see below). This hypothesis is further supported by Woodruff et al., who described pronounced EF B-cell responses at early time points in critically ill patients, and these EF responses produced relatively high levels of nAbs [58]. Of note, although at low titers, nAbs are readily detectable in many patients, suggesting that extensive SHM is not crucial for development of nAbs against SARS-CoV-2 [70, 86, 89, 90]. Consistent with this, early IgA production is an important contributor to the early neutralizing response [72].

Cross-reactive antibodies induced by previous infections with endemic human coronaviruses (hCoVs) were much debated early in the pandemic as potential mediators of cross-protection or induction of antibody-dependent enhancement (ADE) of infection, though in vivo evidence for ADE in coronavirus infections is very limited [91]. Indeed, SARS-CoV-2 reactive antibodies were found to be present in a small proportion of SARS-CoV-2 naïve and unvaccinated individuals, particularly in children and adolescents, but they did not correlate with disease severity in either direction after SARS-CoV-2 infection [29, 92, 93]. These responses mostly involve binding to the more conserved S2 domain of the S protein, and the majority of these antibodies lack neutralizing activity [92, 94–97].

### Mucosal antibody responses

Serum nAb responses induced by vaccination or previous infection correlate with protection against SARS-CoV-2 infection [98–101]. Although such systemic immune responses are easily assessed in blood samples, they do not allow for conclusions to be drawn about local immune responses. Nonetheless, mucosal antibodies and tissue-resident memory T cells play a critical role in the defense against prototypical respiratory viruses such as influenza A virus or respiratory syncytial virus, as shown in preclinical [102–104] and clinical [61, 105, 106] studies.

**Fig. 1:**
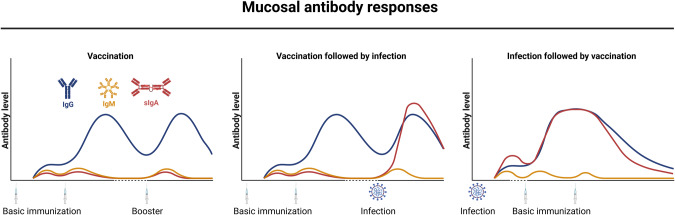
Mucosal antibody responses following infection or vaccination. Systemic SARS-CoV-2 vaccination results in mucosal antibody responses dominated by IgG, whereas IgM and secretory dimeric IgA (sIgA) responses are low and very transient (left). After breakthrough infection, vaccinees display elevated IgG levels and a robust induction of sIgA in the respiratory mucosa (center). SARS-CoV-2 convalescent patients display moderate levels of IgG, IgM, and sIgA responses, and vaccination reinvigorates levels of sIgA and IgG in the respiratory tract (right). Antibody kinetics and relative levels are simplified for ease of interpretation. Created with BioRender.com

At the humoral level, both IgG and secretory dimeric IgA (sIg) may be present in the airway mucosa. Mucosal IgG levels are maintained by neonatal fragment crystallizable (Fc) receptor-mediated transport of systemic IgG across polarized epithelial barriers into luminal secretions [107]. In contrast, secretory IgA is secreted into the luminal space via the polymeric Ig receptor [108] and is thought to depend on tissue-resident MBCs and lung-homing PCs after local antigen exposure via respiratory infection or mucosal vaccination [109]. Consistent with this, SARS-CoV-2 convalescent patients exhibit stronger S- and RBD-specific IgA responses in bronchoalveolar lavage (BAL) and saliva than infection-naïve vaccinees (Fig. 1) [110–112]. Similar to the systemic response, salivary antibodies are readily detectable a few days PSO and peak at approximately two weeks [71, 72]. The early mucosal antibody response consists of IgM, IgA, and IgG, all of which correlate with corresponding serum levels [71], but only mucosal fluids contain dimeric sIgA [72]. In addition, IgA appears to constitute the majority of the mucosal nAb response, at least at early time points PSO [72, 113], but its levels decline more rapidly compared to IgG in mucosal samples. In saliva, anti-S IgA responses return to baseline levels within six months in most patients [111, 114]; other studies have found persistent IgA responses in nasal fluid for more than six and nine months [115, 116]. Use of different sampling and detection methods may be one reason why these studies yielded different results [90].

Wang et al. have shown that monomeric IgA is twofold less potent than IgG in neutralizing SARS-CoV-2 but that dimeric sIgA is 15-fold more potent than IgG [117]. The most likely and straightforward interpretation of these results is increased binding avidity due to the dimeric form. In addition to a higher valency, IgA has other properties that distinguish it from IgG, including the absence of complement activation or ability to engage Fcα receptors [118]. Along these lines, one study showed that nasal IgG is the main correlate of virus phagocytosis and that nasal IgA correlates with virus neutralization [113], supporting the view of some division of labor between different immunoglobulin classes in the airway mucosa. The most intriguing support for the nonredundant functions of IgA is from two studies reporting a positive correlation between mucosal IgA titers and protection against SARS-CoV-2 infection [112, 119]. Havervall et al. examined the risk of breakthrough infection in relation to mucosal IgA and IgG levels in triple-vaccinated health care workers. Individuals with high mucosal IgA levels (>75th percentile) showed a relative risk reduction of 65% compared to 27% for high mucosal IgG levels [112]. For previously infected individuals with high mucosal IgA, the risk reduction was even higher at 79%. Whether IgA acts as a direct COP remains to be investigated. Mucosal IgA may also correlate with the presence of tissue-resident T-cell responses, which may have direct antiviral effects. Such resident T cells are induced at high frequencies in the nasal cavity after SARS-CoV-2 infection and might be directed against several viral antigens [120]. Therefore, it will be important to investigate the contribution of mucosal T-cell and antibody responses to protection by using appropriate sample techniques and study designs, such as nasal immune cell sampling [120].

## B-cell responses to primary infection

Serologic assays remain important for tracking infection- and vaccine-induced immune responses, yet analyses of actual B-cell responses are essential for understanding how certain serologic features develop. Use of state-of-the-art methods to analyze B cells has been a hallmark of the response by the scientific community to the recent pandemic. The knowledge gained will undoubtedly help to optimize vaccine strategies to elicit broadly reactive, long-lasting and protective immune responses.

An important observation was the early appearance of potent nAb clones upon SARS-CoV-2 infection, though this does not necessarily coincide with high serum neutralization titers [70, 86, 89, 90, 121, 122]. One would expect that de novo B-cell responses require time to produce potent nAbs through some degree of affinity maturation, and hCoV-specific MBC responses have been considered a source of such rapidly emerging nAbs. However, despite early recruitment of highly mutated B-cell clones reflecting cross-reactive hCoV memory, these responses are mostly nonneutralizing [92, 94–97] and decline between three and six months after infection, whereas de novo-generated B-cell responses expand and largely contribute to the nAb response [94]. Furthermore, these cross-reactive responses do not appear to confer protection against SARS-CoV-2 [92, 95, 123].

Several studies support the idea that the early nAb response arises de novo from class-switched EF B-cell responses or from GC B cells with limited SHM. B-cell analyses in patients have shown that the infection-induced antibody response is highly polyclonal but that potent neutralizers are close to the germline [23, 28, 124–127] and show enrichment for specific immunoglobulin heavy-chain variable region (IGHV) genes such as 3–53, 1–2, 3–9, and 3–30 [128]. Near germline nAbs may result from the recent zoonotic origin of SARS-CoV-2 and lack of adaptation to the human B-cell repertoire. Ongoing virus evolution in the form of VOCs gradually allows for evasion of these primary immune responses, with the latest omicron variants being able to evade most of the initially described nAb clones [129]. Regardless, it is currently unknown whether omicron variants induce near germline responses with neutralizing activities in SARS-CoV-2 naïve individuals. Such analysis is certainly worthwhile, though finding respective cohorts to study may be difficult.

**Fig. 2:**
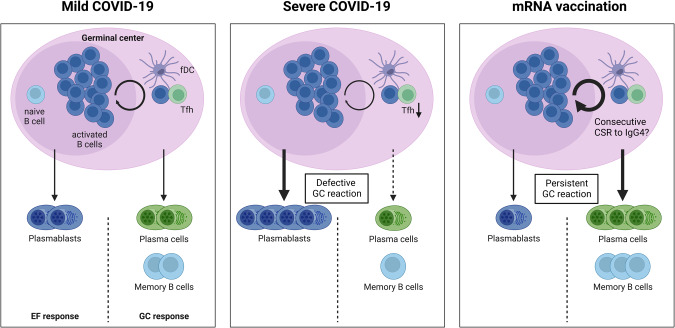
Distinct features of germinal center (GC) reactions after infection or vaccination. Mild COVID-19 leads to extrafollicular (EF) and GC responses (left), whereas severe cases result in defective GC reactions and a shift toward pronounced EF responses (center). In contrast, mRNA vaccines elicit only minimal EF responses but persistent and pronounced GC reactions. Continuous B-cell maturation may cause consecutive class-switch recombination (CSR), which may explain the increased IgG4 levels after repeated mRNA vaccinations. Created with BioRender.com

Another important finding was that severe SARS-CoV-2 infection coincides with exceptionally strong humoral responses, including high nAb titers [58, 88, 130]. At the B-cell level, these serologic findings are paralleled by a significantly decreased frequency of circulating B cells as part of general lymphopenia but a substantial expansion of PBs, accounting for up to 30% of B cells upon acute infection [131–134]. These alterations in the immune system normalize at three to six months after infection [133]. One explanation for the marked shift from B cells to PBs upon acute SARS-CoV-2 infection may be loss of GCs in affected patients. Three studies reported hypoplasia or a lack of GCs in lung-draining lymph nodes, including decreased numbers of FDCs, TFHs, and B cells [135–137]. Kaneko et al. investigated postmortem thoracic lymph nodes and found a dramatic reduction in GC B cells and TFH cells, whereas IgG-producing, class-switched PBs were increased [135]. The authors attributed the lack of TFH cells to increased levels of TNF-α. In the absence of a TFH response, GCs are not induced, and the B-cell response shifts to T-cell-independent EF responses, resulting in PB expansion and exceptionally high antibody responses (Fig. 2).

Despite these EF-like responses early after infection and the absence of GC reactions in critically ill patients, convalescent patients are generally able to mount an MBC evolution over time. Several groups have followed B-cell responses longitudinally and observed an increase in MBCs from early infection to three to eight months PSO, as well as a relatively stable MBC population between six and 12 months after infection [75, 81, 97, 138, 139]. This was true for IgG^+^ but not IgA^+^ and IgM^+^ B cells, which showed a decline from approximately 150 days and 70 days, respectively [75]. S- and especially RBD-specific MBCs exhibited increasing SHM over time, and the corresponding nAbs had higher affinity, potency, resistance to RBD mutations, and neutralization breadth, which explains why neutralization titers remain stable even as RBD IgG levels decline [19, 81, 84, 139–141]. In addition, PCs that produce SARS-CoV-2-specific antibodies remain detectable in the bone marrow for seven to eight months [84]. Therefore, it is tempting to speculate that the biphasic decline in serum IgG reflects an initial, rapid decline in short-lived PBs, followed by a second, slower decline in long-lived PCs. Taken together, these studies suggest an ongoing GC reaction over several months in SARS-CoV-2 convalescent patients, at least after noncritical illness (Fig. 2).

## Antibody responses to SARS-CoV-2 vaccination

Several SARS-CoV-2 vaccines became available in Europe and in the United States between late 2020 and early 2021, including mRNA vaccines and adenoviral vector vaccines. Clinical trials have demonstrated vaccine efficacies against symptomatic disease ranging from 74% to 95%, but the two mRNA vaccines, mRNA-1273 from Moderna (Spikevax) and Bnt162b2 from Pfizer/Biontech (Comirnaty), outperformed the viral vector vaccines in terms of immunogenicity and vaccine efficacy [35–38, 142, 143]. There have also been reports of rare cases of thrombocytopenia induced by adenoviral vector vaccines, mainly AstraZeneca’s ChAdOx-1 vaccine [144–146], leading to restricted use in certain age groups in some countries. As a result, mRNA vaccines dominated vaccination campaigns in the later stages of the pandemic and booster campaigns in Europe and the United States. For these reasons, most of the studies discussed below focus on mRNA vaccine-induced immunity. However, it is important to note that inactivated SARS-CoV-2 vaccines such as CoronaVac (Sinovac Biotech) have been widely used in Asia, South America and other parts of the world. Although the immunogenicity and vaccine effectiveness of CoronaVac against symptomatic infection with ancestral SARS-CoV-2 strains was lower than that of mRNA vaccines, its effectiveness against severe and fatal omicron infection was noninferior [147, 148]. Broader T-cell responses [149] and trained immunity [150] have been proposed as possible mechanisms but are not discussed in this review.

### Basic immunization scheme

Most licensed vaccines were initially licensed for a two-dose schedule. In fact, it quickly became apparent that a single dose of mRNA vaccine did not induce durable antibody responses. nAb responses in vaccinees were reported to peak at approximately 14 days postimmunization, but only 50% actually had detectable nAb titers [142, 151–153]. The same studies demonstrated that a second vaccination resulted in seroconversion and nAb detection in 100% of vaccinees. Accordingly, vaccine effectiveness (VE) against illness was shown to be higher after two doses compared to a single dose, though a single dose still provided almost complete protection against hospitalization for up to 16 weeks [154]. S-specific antibody levels and nAb titers peaked at 14 days after the second vaccination but waned substantially within six months, though nAb titers declined less steeply, probably due to higher antibody maturation at later time points [76, 155–158]. Nevertheless, approximately 16% of twice-vaccinated individuals became seronegative within six months [157].

Due to the varying availability of vaccine doses at the beginning and updated vaccine recommendations, heterologous prime-boost regimens were employed for some vaccinees. Interestingly, heterologous prime-boost vaccination with an adenoviral vector prime followed by mRNA vaccination was superior to homologous vaccine schemes in inducing humoral and cellular immune responses [159–167]. The superior immunogenicity was evident not only in S-specific IgG levels but also in nAb and MBC levels. Similar results were observed for mRNA-1273 and Bnt162b2 as well as for ChAdOx1 and Ad26.COV2-S as primary and secondary vaccine modalities, respectively. The generally lower immunogenicity of homologous ChAdOx1-ChAdOx1 schemes might be explained by interference of anti-vector immunity and the lower immunogenicity of the delivered wild-type S as an antigen. Boosting with 2P-S-encoding mRNA vaccines has been shown to focus the humoral response on prefusion epitopes, resulting in higher and broader nAb responses [163]. Similarly, Ad26.COV2-S encodes a prefusion-stabilized S, but with an additional stabilization at the furin cleavage site [168], resulting in more S1-focused immune responses. Therefore, the Ad26.COV2-S-mRNA scheme consists of two different vaccine antigens, likely resulting in differences in the quantity and quality of humoral responses. In addition, some studies have reported different intervals between homologous and heterologous boosting, which may influence immunogenicity [158, 169].

Convalescent individuals demonstrate a much more efficient response to a primary vaccine dose than those who had not previously been infected. Individuals with previous SARS-CoV-2 exposure exhibited immune responses to a single mRNA vaccine dose comparable to those of uninfected individuals receiving two doses [139, 151, 170]. Notably, convalescent patients showed approximately 1000-fold higher nAb titers, broader neutralization, and resistance to escape mutations than twice-vaccinated noninfected individuals [152]. Nonetheless, infection-induced immunity exhibits significant variation, and a subset of those who have recovered fail to develop humoral immunity. Such individuals respond to an initial mRNA vaccine dose with a kinetic comparable to that of vaccination-naïve individuals [171–173]. A second vaccine dose given within the recommended three- to four-week interval did not further increase humoral or cellular responses in previously infected individuals [158, 174–178]. However, delaying the second vaccination further into later memory or waning immunity led to an efficient boost of antibody responses [158, 169].

### Escape from vaccine-induced humoral immunity

Although the SARS-CoV-2 replication machinery has 3’-5’ exoribonuclease proofreading ability, the virus still exhibits a significant mutation rate of approximately 10^-6^ mutations per nucleotide per replication cycle, which is similar to that of other betacoronaviruses but ten to 100-fold lower than that of other RNA viruses, such as hepatitis C virus or human immunodeficiency virus [179, 180]. This mutation rate allows SARS-CoV-2 to adapt to different selection pressures. On the one hand, the virus adapts to the human host, leading to increased viral fitness and infectivity, as seen with the D614G mutation or the H69/V70 deletion, neither of which provide significant immune escape [181–183]. On the other hand, immune escape mutations became much more prevalent and relevant after large portions of the community had acquired infection- or vaccine-induced immunity against SARS-CoV-2. E484K and N501Y were the first prominent RBD escape mutations that reduced vaccine-induced neutralization of the alpha variant [184, 185]. Immune escape was further observed by the subsequent beta, gamma, and delta variants before the omicron variant with an extensive mutational load in the S gene appeared, leading to exceptional immune escape [180, 186, 187]. The omicron variant and its sublineages BA, BQ, and XBB carry more than 30 amino acid substitutions, insertions, and deletions compared to the ancestral S variant [188, 189]. In light of these developments, vaccine recommendations and booster modalities were repeatedly updated during the pandemic to maintain or increase protective immunity against VOCs.

For pre-omicron variants, a moderate escape from vaccine-induced immunity has been reported [185, 190–193], but two mRNA vaccinations or prior infection plus one vaccination induces robust nAb responses against viral variants [151, 194] and efficient protection from infection [195]. Vaccination with mRNA, viral vector, or inactivated SARS-CoV-2 vaccines induces a broader IgG response compared to infection [196], covering a higher epitope diversity in the RBD [197]. The situation changed with the emergence of omicron variants. These strains efficiently escape from infection- and vaccination-induced immunity as well as from most monoclonal antibodies in clinical use [129, 198–204]. As a result, VE against symptomatic omicron infections and hospitalization also declined after the basic immunization cycle, though the latter remained at a relatively high level [201, 205].

### Booster immunization

A third vaccination with mRNA vaccines encoding the ancestral S variant, the so-called booster vaccination, has been recommended in most European countries and the United States in response to the emergence of omicron variants and waning immune responses in the population. Booster immunization leads to efficient reinvigoration of nAb levels within one week, even exceeding peak levels after the second vaccination [156, 196]. In addition, an mRNA booster dose increases omicron-specific nAb titers [158, 189, 202, 203, 206, 207] and protection against omicron infection to robust levels [201, 205, 208]. Timing of this booster is relevant to vaccination effectiveness in two regards. First, omicron-specific nAb levels [158] and protection [201] decline within six to seven months after the second vaccination, though they are already low at the peak. A third vaccination restores absolute S1-binding antibody quantities similar to the peak response after the second vaccination. Second, booster vaccination promotes further maturation of antibody quality, resulting in higher IgG avidity and improved nAb titers [158]. Interestingly, omicron nAb levels appear to benefit substantially from timely spaced antigen exposures, suggesting diversification of the B-cell response rather than pure selection based on BCR affinity [158]. Boosting such mature B cells in late memory phases may then lead to specific selection of broadly neutralizing clonotypes. However, the resulting cross-reactive antibody responses are transient, waning within six months after both first and even second booster vaccinations [209, 210].

In light of the substantial antigenic distance between the omicron sublineages and the Wuhan-Hu-1-based vaccines, adapted booster vaccines have recently been employed to provide a better vaccine match and to reinvigorate immunity against contemporary variants. Both Moderna and Biontech/Pfizer have released adapted bivalent mRNA vaccines to deliver original+BA.1 or original+BA.4/5 S proteins, which are currently recommended for booster vaccination in European countries and the United States. Davis-Gardner et al. report that a bivalent original+BA.4/5 booster vaccination induces superior nAb levels against the omicron lineages BA, BQ, and XBB compared to one or even two booster vaccinations with the original, monovalent vaccine [39]. Similarly, BA.1–adapted BNT162b2 induced a more efficient boost of BA.1-specific humoral responses than a booster with the original vaccine [40]. It is important to note that the original vaccines also boosted omicron-specific nAb responses, but to a two- to threefold lesser extent compared to bivalent formulations. Two other studies reported a preferential boost in ancestral S-specific humoral immunity and a comparable increase in BA.5-specific immunity by original monovalent and bivalent adapted booster vaccines [211, 212], supporting the idea of antigenic imprinting. Taken together, these immunogenicity studies do not provide conclusive evidence for the absolute superiority of adapted mRNA vaccines, as both vaccines induce omicron nAbs at similar or only moderately different levels. The most intriguing argument in favor of adapted vaccines is from real-world estimates of booster effectiveness. A retrospective matched cohort study showed that the bivalent BA.4/5 mRNA vaccine provided better protection against severe BA.5 infection than a booster with the original mRNA vaccine [42]. Specifically, the VE of the bivalent original+BA.4/5 was estimated at 51.0%, and the VE of the original+BA.1 and monovalent ancestral vaccines was estimated at 49% and 27%, respectively. Another study concluded that VE against hospitalization was 25.2% for the monovalent booster and 58.7% for the bivalent booster, with 24.9% and 61.8% against death after a monovalent or bivalent booster, respectively [41]. Whether this protection is due to increased induction of omicron-specific nAbs remains to be investigated, as there is also evidence that T-cell responses contribute to viral clearance in breakthrough infections [213, 214].

### Mucosal immune responses

SARS-CoV-2 vaccines are primarily evaluated by induction of systemic nAb responses, but mucosal immune responses may also contribute to protection against infection. S-specific IgG is induced in saliva samples after vaccination but at 100-fold lower levels compared to serum levels [196, 215]. In contrast, salivary IgA is not detectable or only at low levels after two or three vaccinations (Fig. 1) [215, 216]. Importantly, the beneficial effect of a third vaccination to induce omicron neutralization in serum is not reflected in saliva samples. Indeed, only 4% of vaccinees show a detectable nAb response after the third mRNA vaccination [216]. As mentioned above, induction of mucosal sIgA is particularly dependent on local antigenic stimuli, and therefore, breakthrough infection after two or three doses of mRNA vaccine results in substantially higher salivary IgA levels than in vacinees without breakthrough infection [112, 119]. In addition, Park et al. reported that omicron breakthrough infection, but not vaccination alone, induced nAb responses in nasal secretions capable of neutralizing omicron and the ancestral SARS-CoV-2 strain [193]. These hybrid nAb responses peak at approximately two to three weeks postinfection and remain detectable for more than 180 days. Importantly, infection-primed mucosal antibody responses can be reinvigorated by parental SARS-CoV-2 vaccination (Fig. 1) [217]. These findings suggest that systemic mRNA vaccination may not be very efficient in establishing mucosal B-cell responses de novo but may be an option to boost anamnestic responses. One explanation may be that tissue-resident B-cell responses induced by previous SARS-CoV-2 infection [218] are reactivated by circulating antigen produced after systemic mRNA vaccination [219] but that the circulating antigen levels are insufficient to initiate mucosal MBCs.

## B-cell responses to SARS-CoV-2 vaccination

### B-cell response dynamics

S protein-specific PB responses peak at approximately two weeks after primary mRNA vaccination and at approximately one week after a second mRNA dose before declining significantly to background levels within three to four weeks in most individuals [220, 221]. Fine-needle aspiration of lymph nodes has allowed for analysis of vaccine-induced GC B-cell dynamics. Turner et al. reported that levels of S-specific GC B cells from draining axillary lymph nodes were stably maintained for at least 12 weeks after a second vaccination [220]. Such long-lasting GC reactions were not observed after influenza vaccination in humans [222] or after alum-adjuvanted protein immunization in mice [223]. Congruent findings were also reported for TFH responses, which persisted at robust levels for at least six months after mRNA vaccination and showed a significant correlation with GC B-cell responses [224]. Consistent with these observations, mRNA vaccination, but not MF59-adjuvanted protein vaccination, induces potent GC reactions in mice that correlate with an increased TFH response, nAb production, and differentiation of long-lived PCs and MBCs [225]. A consequence of ongoing GC responses is a progressive increase in SHM in MBCs and emergence of nAbs with increased affinity and neutralization of derived nAbs [226]. Thus, mRNA vaccines promote strong and durable GC reactions consisting of persistent GC B and TFH populations in draining lymph nodes, eventually resulting in nAbs with progressively increasing affinity (Fig. 2). The exact mechanisms of the GC-stimulating effects of mRNA vaccines remain to be investigated but might be related to intrinsic adjuvant effects of the lipid nanoparticles or their systemic trafficking [227, 228]. Furthermore, a logical prerequisite for such ongoing GC reactions is persistence of the respective antigen, allowing for selection of affinity-increased B cells. Röltgen et al. confirmed this hypothesis by detecting vaccine mRNA and S protein in the lymph nodes of vaccinated but not previously infected individuals for up to 60 days [196]. Vaccine antigen was detected around GC foci, indicating ongoing antigen presentation by follicular DCs.

### B-cell responses in hybrid immunity

By the time the vaccination campaigns began, some individuals had already been infected with SARS-CoV-2, and others experienced breakthrough infections after vaccination. These and even more complicated histories of antigen exposure create “hybrid immunity”. Prior SARS-CoV-2 infection enhances the immunogenicity of primary SARS-CoV-2 vaccination, resulting in a humoral response amplitude comparable to that of two doses of mRNA vaccine in infection-naïve individuals [139, 151, 170]. The level of infection-derived B-cell memory is predictive of the postvaccination response in immunized convalescent patients [229]. Such hybrid immunity also provides broader cross-variant neutralization by targeting more conserved parts or by broadly inducing nAbs through extensive SHM [139, 230, 231]. As described above, omicron evades vaccination- or previous variant infection-induced immunity [232–234]. As a consequence of this antigen mismatch, primary infection with an omicron variant mostly induces omicron sublineage-specific immunity in immunologically naïve individuals, which in turn neutralizes earlier variants poorly. Interestingly, preimmune individuals in whom non-omicron immunity is mounted and who experience omicron breakthrough, particularly BA.4 or BA.5 infection, show broad neutralization across all tested variants. Such breakthrough infection primarily activates the MBCs that recognize conserved epitopes between ancestral and omicron lineages rather than promoting de novo B-cell responses [193, 235]. Although such immune imprinting appears to be beneficial for induction of broadly reactive antibodies against known variants, it hinders induction of neutralizing responses against novel epitopes. This may be detrimental if conserved sites escape from immunity in newly emerging variants.

Several more mechanisms are discussed below as explanations for the beneficial features of hybrid immunity. First, vaccine- and infection-primed B-cell responses use different variable region genes, i.e., select different B-cell clones, which might broadly favor nAbs in convalescent patients [236, 237]. Second, SARS-CoV-2 infection induces more MBCs and more pronounced T_H_1 responses than vaccination [238]. An altered CD4^+^ T-cell response, especially TFHs, may also contribute to altered GC reactions and B-cell responses, as has been shown in critically ill patients [135]. Third, antibodies generated by the primary response directly modulate secondary responses against SARS-CoV-2 by blocking or enhancing recruitment of naïve B cells [239]. Infection- and vaccination-induced antibody responses may therefore differentially modulate secondary B-cell responses. Fourth, regardless of the actual immunization modality, the timing of repeated antigen contacts directly influences the characteristics of the immune responses provoked. Several studies suggest that timely spaced antigen contacts promote more extensive GC reactions compared to shorter vaccination intervals, resulting in high affinity and cross-reactive nAbs [158, 169, 240]. This effect was also observed with extended intervals between mRNA doses, independent of an infection event [241]. Therefore, the strict three-week interval between the first two mRNA doses may induce a less efficient immune response compared to a single vaccination followed by SARS-CoV-2 infection with longer intervals between antigen exposures. Ultimately, hybrid immunity is more durable and provides the most efficient protection against infection. Goldberg and colleagues showed that at six to eight months after the last antigen exposure, the risk of infection is approximately four to five times lower with hybrid immunity compared with immunity after two or three doses of mRNA vaccine [242]. Further studies are needed to define protective correlates in hybrid immunity, as it consists of more immune parameters than nAbs. Nonneutralizing Abs against viral proteins other than S, T-cell responses, and mucosal immunity may all contribute to the beneficial effects observed with hybrid immunity.

### Unusual class switching toward IgG4 after mRNA vaccination

nAb responses are an important COP against SARS-CoV-2 [98]. However, there is accumulating evidence from other viral infections that the neutralizing capacity is not the only function that is important for the antiviral activity of antibodies. For example, Fc functions critically contribute to antibody-mediated protection from Ebola infection [243], and broadly neutralizing hemagglutinin stalk-specific antibodies require Fcγ receptor interactions for protection against influenza [244].

Vaccination with one or two doses of mRNA vaccine induces a humoral response dominated by IgG1 and IgG3 subclass antibodies [245]. These two IgG subclasses mediate prototypical Fc effector functions such as antibody-dependent cytotoxicity, antibody-dependent phagocytosis, or antibody-dependent complement activation, even against the omicron variant that escapes their neutralizing activity. However, in a longitudinal study, we observed that the proportion of S-specific IgG4 increased significantly at approximately six months after the second and even more after the third mRNA vaccine dose [246]. S-specific IgG4 was undetectable in most vaccinees after the first and early after the second vaccination but reached almost 20% of the IgG response late after the third vaccination. Up to 37% of the S-specific MBC response consisted of IgG4^+^ MBCs. IgG4 is a noninflammatory IgG subclass with poor Fc effector functions [247]. Concomitantly, sera containing high levels of IgG4 provided few antibody-mediated effector functions, such as antibody-dependent phagocytosis or complement deposition, despite increased antibody affinity and neutralizing capacity in our study. Importantly, total IgG4 levels in the sera of vaccinees did not increase after mRNA vaccination (our own unpublished observation).

Buhre et al. confirmed a shift toward IgG4 late after the second mRNA boost with the mRNA-1273 and BNT162b2 vaccines but not after two vaccinations with viral vector vaccines [248]. Interestingly, they also reported a stronger IgG4 induction by mRNA-1273 compared to BNT162b2, in line with the slightly lower immunogenicity of the latter [245, 249]. Therefore, both studies link the profound induction of S-specific IgG4 with repeated vaccination with mRNA lipid nanoparticle vaccines. Similar to our own findings, elevated anti-S IgG4 antibodies were not found when vector-based vaccines were used for primary vaccination [250], and elevated IgG4 anti-S levels were also not common in subjects infected with SARS-CoV-2 prior to vaccination [250]. It appears that processes following mRNA vaccination in the priming phase of the immune response drive the class switch to distal isotypes.

**Fig. 3:**
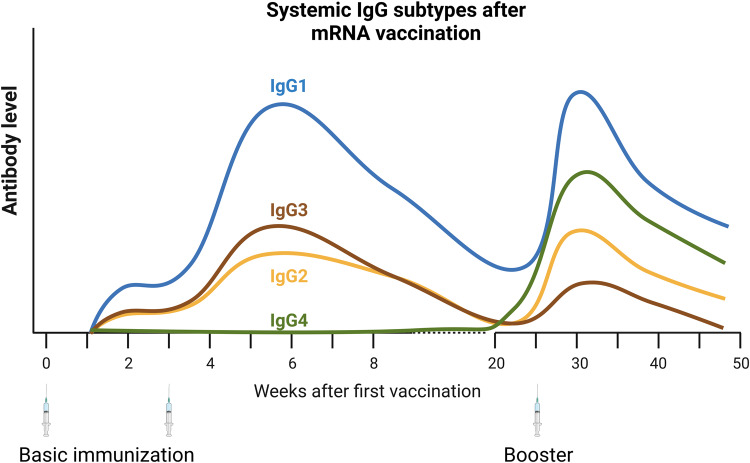
Systemic IgG subtype kinetics following vaccination. S-specific humoral responses induced by the basic immunization cycle with mRNA vaccines are dominated by IgG1 and IgG3, which possess proinflammatory Fc functionalities. Late after the second vaccination, and especially after the booster dose, IgG1 is still dominant, but the fraction of noninflammatory IgG3 and IgG4 is substantially increased. Antibody kinetics and relative levels are simplified for ease of interpretation. Created with BioRender.com

High IgG4 levels have mostly been observed in the context of repeated antigen contacts, such as successful allergen-specific immunotherapy, and in beekeepers after several beekeeping seasons [251–253]. These studies describe a slow rise in IgG4 levels after multiple antigen exposures; other IgG subclasses were readily measured at early time points. It is tempting to speculate that IgG4 class-switched B cells emerge after consecutive class-switch recombination events. Of the four γ heavy chain genes (γ3-γ1-γ2-γ4), γ4 is the most downstream. Therefore, IgG4 class-switched MBCs may require multiple antigen contacts and an extensive GC reaction to develop [254]. This idea fits well with mRNA vaccines, which induce persistent GC and TFH responses (Fig. 3) [220, 224]. Another possibility is that vaccine-induced, freely circulating S antigen stimulates MBCs in the absence of inflammatory costimulants [196]. Such a situation would be similar to allergen immunotherapy, whereby repeated low-dose antigen administration in the absence of inflammation results in antigen-blocking IgG4. However, the exact mechanisms of mRNA vaccine-induced IgG4 need to be investigated with high priority. Due to the different organization of the IgH gene locus and the different regulation of class switch recombination, studies in mice may not be adequate. Interestingly, nonhuman primates show similar or even more pronounced IgG4 responses after an immunization regime with the mRNA-1273 vaccine predominantly used in humans during the pandemic, i.e., immunizations at weeks 0 and 4 and boosters months later [255]. Therefore, injection of the second highly potent antigen dose shortly after the first, presumably during the ongoing GC response, may be responsible for the unusual class switch toward downstream γ heavy-chain genes.

Importantly, despite reduced engagement of Fc-mediated effector functions, the increase in IgG4, as expected, does not negatively impact in vitro neutralizing capacity. This is consistent with reports indicating that mRNA boosters increase protection against infection [41, 42]. However, due to the high selective pressure on neutralizing epitopes in the RBD, VOCs often show immune escape at these sites. As up to 95% of S-specific antibodies are nonneutralizing [256–258], there may be a shift in the predominant antiviral function from neutralization to Fc-mediated functions against future immune escape variants. In such a scenario, a substantial fraction of IgG4 would provide less antiviral functions. Along these lines, omicron variants have been shown to escape the RBD-specific nAb response [259, 260], whereas nonneutralizing responses recognizing other parts of S retain antigen- and Fcγ receptor-binding activity [256]. At least in preclinical studies, Fcγ receptor-dependent antibody effector functions are essential for mRNA vaccine-mediated protection [261]. In the context of human SARS-CoV-2 infection, IgG4 has not been well explored, but two studies do describe circumstantial observations. In one, elevated total IgG4 in sera predicted higher lethality in COVID-19 patients [262]. This may be related to the premorbidity of these patients, as high IgG4 levels are a feature of IgG4-related disease, an inflammatory disease of unknown origin [263]. Additionally, elevated S-specific IgG4 antibodies were observed early in infected patients who were more likely to die from COVID-19 [264]. IgG4 was still a minor contributor to all anti-S antibodies in that study, suggesting confounding effects in this cohort. It is important not to draw false or premature conclusions from these studies. mRNA vaccination, especially booster vaccination leading to high IgG4 levels, clearly demonstrates robust VE. Nevertheless, the mechanism behind and the consequences of repeated mRNA vaccination-induced IgG4 remain unclear and require further investigation.

## Concluding remarks and perspectives

The recent SARS-CoV-2 pandemic has had a dramatic impact on several aspects of life, including social interaction, the economy, and health care. At the same time, the research community initiated an unprecedented research effort that ultimately led to key insights in immunology, virology, and vaccinology. B-cell and antibody responses were at the center of this research because of their strong association with protective immunity. Similar to the important insights generated by HIV research over more than three decades, SARS-CoV-2-related research will shape human immunology and vaccine design for decades.

Novel vaccine platforms, including mRNA, viral vector, and recombinant protein vaccines, were developed at “light speed” and employed in exceptionally large cohorts. Moreover, use of state-of-the-art technologies allowed for in-depth analyses of vaccine-induced immunity. In particular, mRNA vaccine technology demonstrated impressive immunogenicity but also revealed some surprising features, such as persistent GC reactions and unusual induction of IgG4 antibodies. These phenomena should be further explored in future research, especially as mRNA vaccines are being developed for other infectious diseases.

Although vaccines are critical and effective in controlling the pandemic phase, emergence of escape variants, including the omicron complex, limits VE and demands vaccine adaptations. Now that we are likely to approach an endemic situation with newly emerging virus variants each season, similar to influenza A drift variants, it is desirable to maintain stable protective immunity over several seasons. This applies on the one hand to the amplitude and persistence of vaccine-induced immunity and on the other hand to the specificity of the immune response, which should target conserved sites of the S protein. Optimally, vaccine strategies should establish such durable and broadly reactive immune responses to protect persons at risk over several seasons. The minimal goal is to regularly vaccinate these individuals with adapted vaccines, as is currently the case for adapted mRNA boosters. Regardless, the difficulty in predicting the dominant virus variants in the upcoming season and the inherent risk of vaccine mismatches is evident in seasonal influenza vaccinations.

An additional feature of mRNA and viral vector vaccines is efficient induction of T-cell immunity and not only humoral responses. nAbs correlate with protection from infection, but future studies should also investigate the contribution of other adaptive immune responses to protection, such as nonneutralizing antibodies and T cells. Both immune parameters recognize epitopes that appear to be more conserved among SARS-CoV-2 variants and might become important COPs in the case of strong immune escape of nAb targets.

Mucosal immune responses have also gained attention as potential COPs. sIgA and tissue-resident memory T cells are not efficiently established by current SARS-CoV-2 vaccines but instead require mucosal antigen delivery. Therefore, harnessing mucosal immunity through vaccination will require intensive research on mucosal vaccine platforms. It will be crucial to develop strategies that induce strong but also durable immune responses in the respiratory tract. In addition, there is a lack of non- or minimally invasive routine methods to sample these immune responses in different parts of the respiratory tract, especially for T-cell analyses. These methods and appropriate study designs will be essential to define mucosal COPs and evaluate mucosal vaccines in clinical trials.
